# Correlation between Visual Functions and Retinal Morphology in Eyes with Early and Intermediate Age-Related Macular Degeneration

**DOI:** 10.3390/ijerph17176379

**Published:** 2020-09-02

**Authors:** Rituparna Ghoshal, Sharanjeet Sharanjeet-Kaur, Norliza Mohamad Fadzil, Haliza Abdul Mutalib, Somnath Ghosh, Nor Fariza Ngah, Roslin Azni Abd Aziz

**Affiliations:** 1 Optometry and Vision Science Program, Faculty of Health Sciences, University Kebangsaan Malaysia, Jalan Raja Muda Abdul Aziz, Kuala Lumpur 50300, Malaysia; rituparna4ab@yahoo.co.in (R.G.); norlizafadzil@ukm.edu.my (N.M.F.); halizamutalib@ukm.edu.my (H.A.M.); 2 Department of Optometry, Supreme Institute of Management and Technology, Mankundu Hooghly 712123, West Bengal, India; 3 Department of Allied Health Sciences, Brainware University, Barasat, Kalkata 700125, West Bengal, India; somnath4ab@yahoo.co.in; 4 Department of Ophthalmology, Hospital Shah Alam, Persiaran Kayangan, Seksyen 7, Shah Alam 40000, Selangor, Malaysia; drfarizangah@gmail.com (N.F.N.); roslinazni@gmail.com (R.A.A.A.)

**Keywords:** early and intermediate ARMD, retinal layers morphology, visual functions

## Abstract

In early and intermediate age related macular degeneration (ARMD), visual acuity alone has failed to explain the complete variation of vision. The aim of the present study was to determine correlation between different visual functions and retinal morphology in eyes with early and intermediate ARMD. In this single center cross sectional study, patients diagnosed as early or intermediate ARMD in at least one eye were recruited. Visual functions measured were best- corrected distance visual acuity (DVA), near vision acuity (NVA), reading speed (RS), and contrast sensitivity (CS). Parameters such as thickness (RT) and volume (RV) of the retina, outer retinal layer thickness (ORLT) and volume (ORLV), outer nuclear layer thickness (ONLT) and volume (ONLV), retinal pigment epithelium layer-Bruch’s membrane complex thickness (RPET) and volume (RPEV) were assessed employing semi-auto segmentation method of Spectralis optical coherence tomography (OCT). Twenty-six eyes were evaluated. DVA, CS, and RS showed significantly good correlation with RPET, ONLT, and ONLV, whereas NVA showed good correlation with ONLV and RPET. The present study concluded that RS, CS, NVA, and DVA represent the morphological alteration in early stages and should be tested in clinical settings. ONLT, ONLV, and RPET morphological parameters can be employed as important biomarkers in diagnosis of early to intermediate ARMD.

## 1. Introduction

Age related macular degeneration (ARMD) is a chronic degenerative condition of the retina that leads to significant visual impairment in elderly population. By 2020, the projected number of ARMD worldwide is 196 million [1]. Early and intermediate stages of ARMD are characterized by deposition of drusen between retinal pigment epithelium (RPE) and Bruch’s membrane (BM) and/or alteration of retinal pigment epithelium in the macular area, whereas in later stages, the disease may progress to either geographic atrophy (GA) or neovascular ARMD (n-ARMD) [2,3]. Although there are different treatment options available for n-ARMD, cure for ARMD continues to be elusive. Continuous research effort is made to prevent the further progression of early and intermediate ARMD to late ARMD [4].

Introduction of optical coherence tomography (OCT) has revolutionized the in-vivo cross sectional examination of retinal diseases including ARMD, which was previously only possible in histological studies. In recent years, alterations in retinal layers’ morphology in early ARMD has been studied in detail to investigate the disease pathology, which is yet to be fully implicit. While, retinal pigment epithelium-Bruch’s membrane (RPE-BM) complex is reported to be significantly thicker in early and intermediate ARMD compared to normal controls [5], significant thinning of RPE/photoreceptor (PR) and outer nuclear layer (ONL) over drusen was observed [6,7]. Inner retinal layers including the retinal nerve fiber layer (RNFL) and ganglion cell layers (GCLs) are reported to be well preserved in the central fovea and parafovea, despite some alteration in outer retinal layer in early to intermediate ARMD [8]. However, with the increased severity of the disease, the inner plexiform layer becomes thinned [8]. The OCT findings are consistent with histopathological findings in ARMD that reported Bruch’s membrane (BM), retinal pigment epithelium (RPE) and photoreceptors are the structures that are primarily affected [9,10]. However, association of these morphological changes with the visual functions of early and intermediate ARMD eyes is not yet well established. Relationship between morphological changes seen in OCT and visual functions in early and intermediate ARMD will guide the clinician in finding out the validated morphological and visual parameters that are mostly affected by the disease process endorsing surrogate endpoint for clinical trials seeking management for dry ARMD. Furthermore, suitable morphological and visual parameters will assist in the detection, treatment, and long-term monitoring of this chronic degenerative disease. While drawing this correlation in a retrospective study, Pappuru et al. reported inner segment-outer segment of photoreceptor (IS-OS), and ONL to be moderately associated with high contrast distance visual acuity [11]. Furthermore, Karampelas et al. reported a significantly thicker mean RPE-BM complex in early and intermediate ARMD [5]. In this retrospective research, distance visual acuity measured with Snellen’s chart did not achieve a good correlation with RPE-BM complex (*r* = −0.363 in central early treatment diabetic retinopathy study (ETDRS) subfield). Thus, both the studies found moderate correlation of selected OCT parameters with high contrast visual acuity that failed to explain the complete variation of vision, making the parameters less suitable to use as biomarker in early and intermediate ARMD. However, neither of the two studies considered other visual functions such as contrast sensitivity, near acuity or reading speed while drawing these correlations. High contrast visual acuity alone may not always provide complete information of visual damage caused by the disease. High contrast visual acuity may often miss the visual damage in many instances that are picked by contrast sensitivity and reading speed measurements [12,13,14,15].

Therefore, the objective of the present study was to determine the correlation between different visual functions and outer retinal layers thickness in eyes with early and intermediate ARMD. The outcome of the study could identify visual functions other than visual acuity that can reflect macular integrity as a whole, as well as provide correlation between the visual functions and altered retinal morphological parameters.

## 2. Materials and Methods

### 2.1. Study Design and Participants

A single center cross-sectional observational study was conducted in an Ophthalmology Clinic of a public hospital of Malaysia, which is also an ARMD referral center. Patients diagnosed as early or intermediate ARMD in at least one eye were recruited for the study between December 2016 and July 2017. Eyes with medium sized drusen of more than 63 microns was considered as early ARMD, whereas eyes with at least one large drusen of >125 micron and/or any pigmentary abnormalities of the retina was considered as intermediate ARMD [3]. Lesions were assessed in the two-disc diameter of fovea in the study eyes. Eyes with any other retinal pathology including GA, n-ARMD, media opacity such as significant cataract, corneal opacity, glaucoma, amblyopia, or any neurological disease that may affect the normal visual functions were excluded.

Sample size estimation for single proportion for large population formula was used to calculate the sample size [16]. The *p* value of 0.07 was taken from a study by Cheung et al. [17]. The 95% confidence level *Z* value used is 1.96 and precision, Δ^2^ is 10%.

$$n=\frac{{\left(Z_{\alpha /2}\right)}^{2}p(1-p)}{\Delta^{2}}=\frac{{\left(1.96\right)}^{2}\times 0.07\times (1-0.07)}{0.1^{2}}=25.0086=25$$

Therefore, the sample size for the study is 25 patients.

Ethical approval was obtained from Medical Research and Ethics Committee of National Medical Research Register (NMRR-16-1965-31826 (IIR)) and Universiti Kebangsaan Malaysia Research and Ethics Committee (UKM 1.5.3.5/244/NN-186-2014), which follows the tenants of Helsinki. Written consent was obtained from all the participants.

### 2.2. Visual Functions

A 4-m early treatment diabetic retinopathy study (ETDRS) chart was used to measure the best- corrected distance visual acuity (DVA). Pelli-Robson chart was used at 1 m to measure the contrast sensitivity (CS). CS was recorded based on the contrast of last triplet in which two or three letters were correctly read. A standard illumination of 85 candela per square meter (measured using ISO Tech Digital Luxmeter) was maintained during the CS assessment. Near visual acuity (NVA) and reading speed (RS) were recorded using a Universiti Teknologi MARA (UiTM) Malay related-word reading chart [18]. Subjects were instructed to read the sentences loudly from the first line as fast as possible. They were asked to continue reading towards smallest sentences until they could no longer manage to read any of the words in a particular line or examiner had instructed them to stop reading. NVA was recorded as the threshold or the smallest print size that was read correctly by the patient with best spectacle correction whereas reading speed was recorded by dividing the number of words that were read correctly with the time taken to read in words per minute. Mistake or omission of words was noted.

### 2.3. Retinal Morphology

In the present study, Spectral Domain OCT (Spectralis Heidelberg Retina Angiograph + Optical Coherence Tomography (HRA+OCT) Heidelberg Engineering Inc., Heidelberg, Germany) was used to evaluate the retinal morphology of the study eyes. OCT was performed on dilated pupil to achieve best images. The number of B-scan obtained in each examination was 25 with a pattern size of 30 × 20 degree and the distance between B-scan was 242 micron. Auto segmentation mode was activated in all the examination.

### 2.4. Image Analysis

An incorporated auto-segmentation method of retinal layers was used to measure the specific layers of interest. However, manual correction was done in case of any misalignment of segmentation lines with the predefined layer of interest. In OCT thickness map, the layers of interest were selected one by one to measure the thickness and volume of the particular layer. Here, it is important to note that total retinal thickness was measured from internal limiting membrane to Bruch’s membrane (BM) as Spectralis software always set the BM line as the outer retina threshold border.

Average thickness and volume of the following parameters at central 1 mm of the ETDRS grid were chosen for analysis:

- total retina (RT): distance between internal limiting membrane and Bruch membrane;
- retinal outer segment (ORT) or RPE-photoreceptor complex: distance between external limiting membrane and Bruch membrane;
- retinal pigment epithelium-Bruch membrane complex (RPET): distance between retinal pigment epithelium and Bruch membrane;
- outer nuclear layer (ONL): distance between outer layer of outer plexiform layer and external limiting membrane.

**Figure 1:**
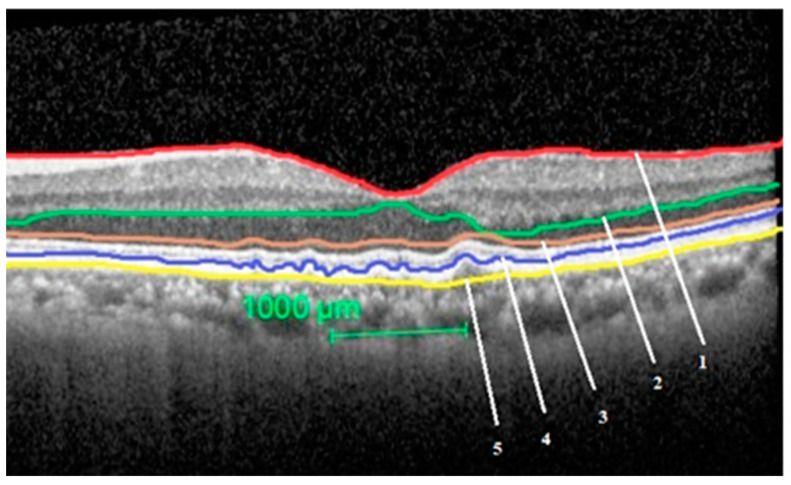
Segmentation of the study layers in an eye with intermediate age related macular degeneration (ARMD). Total retinal thickness = between layer 1 and 5, outer nuclear layer thickness = between layer 2 and 3, outer retinal layer thickness = between layer 3 and 5, retinal pigment epithelium-Bruch’s membrane (BM) complex thickness = between layer 4 and 5.

The segmentation of the layers performed in an eye with intermediate ARMD is shown in Figure 1.

### 2.5. Examiners

Two examiners participated in an inter-observer study where one examiner (Examiner 1, RG) had experience in analyzing the OCT images manually and automatically. The other examiner (Examiner 2, RS) was an experienced retina specialist who had ample experience in all retinal diagnostics including OCT. While analyzing the OCT images, the examiners were masked about the other clinical details of the patients including fundus findings and visual functions. They independently analyzed all the images at different times to ensure no discussion on the image analysis was made.

### 2.6. Statistical Analysis

Normality of the data was assessed using Shapiro–Wilk test. Descriptive statistics was used to report the visual functions and OCT parameters. Pearson’s correlations were used to evaluate the correlation between visual function and OCT parameters. In case of non-normal data, Spearman’s correlation was used. Intra class correlation was employed to assess the inter observer variability of the retinal layer’s thickness.

## 3. Results

Twenty-six eyes diagnosed with mild to intermediate ARMD of 25 patients aged between 51 to 85 years were evaluated in the study. Among 26 eyes, seven eyes were mild ARMD whereas 19 eyes were intermediate ARMD. The demographic characteristics of the study subjects are described in Table 1. Analysis of visual functions and retinal morphology was done by combining early and intermediate ARMD, due to both early and intermediate ARMD comes under dry form of ARMD, and also due to the small number of early ARMD subjects.

### 3.1. Visual Functions

Among all 26 eyes examined, 13 eyes had distance visual acuity of 0.3 logMAR or better. Eighteen eyes had near visual acuity of 0.3 logMAR or better. Nineteen eyes had contrast sensitivity of 1.2 log contrast or better. Fourteen eyes had reading speed of 100 words per minute or better. However, the visual functions varied in a wide range causing mild to moderate impairment. While segregating the visual functions of early and intermediate ARMD eyes, it has been observed that all the visual functions in early ARMD were near normal. However, eyes with intermediate ARMD indicated towards impairment of all the visual functions. Visual functions of the study eyes are described in Table 2.

### 3.2. Retinal Morphology

**Figure 2:**
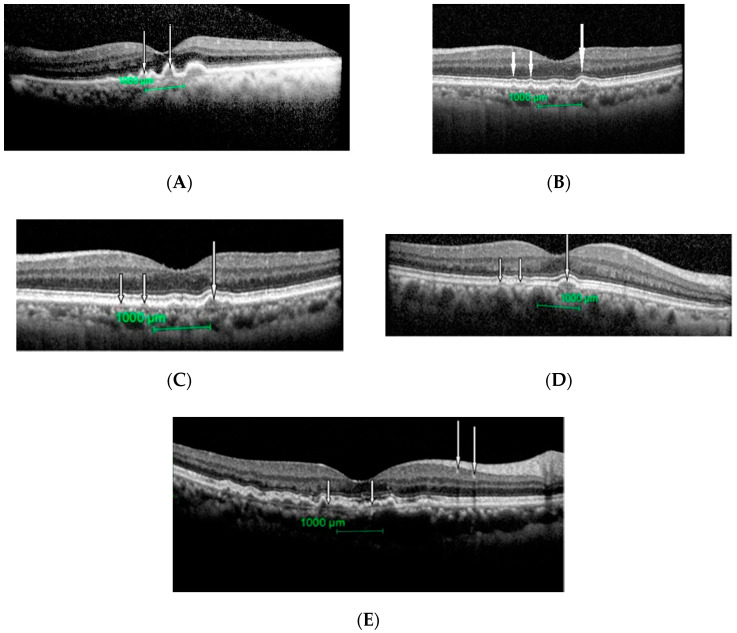
Different size and texture of drusens seen in patients with intermediate ARMD. (**A**): Drusen can be seen as dome-like elevation of the retinal pigment epithelium protruding into adjacent photoreceptor layers (white arrows). (**B**): Cluster of drusens (large arrows) around fovea with a hyper-reflective material (small arrow). (**C**): Few small (small arrows) and a large (large arrow) drusen. External limiting membrane and photoreceptor inner and outer segment junctions are intact here. (**D**): Few medium sizes (small arrows) and a large (large arrow) drusen. External limiting membrane and photoreceptor inner and outer segment junctions are intact here. (**E**): Extensive large drusens with hyper-reflective materials seen (white arrows).

All eyes had drusens with different texture as shown in Figure 2A–E.

Inter-observer reliability was high for all measured OCT parameters, including average retinal thickness (intra class correlation = 0.98), average retinal volume (intra class correlation = 0.89), average outer retinal layer thickness (intra class correlation = 0.75), average outer retinal volume (intra class correlation = 0.90), average outer nuclear layer thickness (intra class correlation = 0.92), average outer nuclear layer volume (intra class correlation = 0.89), average retinal pigment epithelium-Bruch membrane thickness (intra class correlation = 0.79), average retinal pigment epithelium volume (intra class correlation = 0.94). Mean OCT parameters of eyes with early and intermediate ARMD eyes are shown in Table 3. Furthermore, in the present study, there were no noticeable difference found in the thickness and volume of total retina, outer retinal layer between early and intermediate ARMD. However, thickness and volume of retinal pigment epithelium-Bruch membrane complex and outer nuclear layer between early and intermediate ARMD were significantly different (*p* < 0.05).

### 3.3. Correlation between Visual Functions with Retinal Morphology

Correlation between visual functions and retinal morphology were assessed in eyes with early and intermediate ARMD. As done in previous research [5], early and intermediate ARMD were grouped together while drawing correlation between visual functions and retinal morphology. This is well justified, as both early and intermediate ARMD comes under dry form of ARMD and shares similar morphological changes that differ in intensity or severity.

Distance visual acuity, contrast sensitivity and reading speed showed significantly good correlation with average retinal pigment epithelium thickness, average outer nuclear layer thickness and volume (*r* ≥ 0.5, *p* < 0.05) whereas near visual acuity showed a good correlation with average outer nuclear layer volume and average retinal pigment epithelium thickness (*r* ≥ 0.50, *p* < 0.05). A modest correlation was observed between near visual acuity & average outer nuclear layer thickness (*r* = −0.45, *p* < 0.05) and between contrast sensitivity & average retinal pigment epithelium volume (*r* = −0.43, *p* < 0.05). However, total retinal thickness and outer retinal thickness did not show any significant correlation with any of the visual functions (*p* > 0.05). The correlation is described in Table 4. Furthermore, while drawing association between OCT parameters, a strong negative correlation (*r* = −0.62, *p* < 0.01) was found between average retinal pigment epithelium-Bruch membrane thickness and average outer nuclear layer thickness. However, there were no significant correlation (*p* > 0.05) found between the other OCT parameters measured in this study.

## 4. Discussion

The present study showed that in early ARMD, the visual functions may remain near normal, keeping correspondence to the minimal morphological changes However, in intermediate ARMD, the morphological changes vary from large drusen to pigmentary changes in the retina [2,3]. This resulted into some variation in visual functions between early and intermediate ARMD. Difference in the average retinal pigment epithelium thickness and average outer nuclear layer thickness found between the early and intermediate ARMD eyes in the present study may further support these findings.

Visual acuity of early to intermediate ARMD is reported in a wide range of variations in past researches. Difference in study populations might have attributed to these differences. Zhichao et al. reported an average VA of −0.03 ± 0.09 logMAR with a range of −0.22 logMAR to 0.16 logMAR in 100 eyes with intermediate ARMD [19]. However, major aim of this study was to determine the relationship between night vision symptoms and visual function measures in intermediate ARMD. Thus, patients with distance visual acuity of less than 0.3 were excluded in the above study. Patel et al. reported a distance visual acuity between 0.06 to 0.44 in 67 eyes with early to intermediate ARMD [20]. Conversely, Pappuru et al. reported a distance visual acuity range between 1.3 to 0.00 in 100 eyes with dry ARMD, including geographic atrophy (GA) [11]. Likewise, another longitudinal study reported a baseline mean distance visual acuity of 0.093 ± 0.137 with a range of −0.080 to 0.460 in 12 eyes with intermediate ARMD. When they were followed for a period of 6 years, the mean visual acuity was recorded as 0.492 ± 0.566, with a range of 0.000 to 1.600 logMAR, where 2 among the 12 eyes had converted to GA [21].

However, high contrast distance visual acuity is not always well correlated with morphological parameters and quality of life in diseased eyes especially in macular diseases [12,14,15]. Therefore, the assessment of other visual parameters, such as contrast sensitivity and reading ability, may better reflect macular integrity as a whole, and thus provide stronger correlations with OCT-derived morphologic parameters in ARMD [14,22]. Besides, contrast sensitivity that cannot be predicted by high contrast visual acuity, sometimes provides more robust information on visual functions [23,24]. Although contrast sensitivity was suggested to be incorporated in clinical evaluation while monitoring and treating ARMD patients [14,20,22], not many studies reported contrast sensitivity of dry ARMD eyes. Patel et al. reported a mean contrast sensitivity of 27 letters (i.e., approximately 1.35 log contrast) and 24 letters (i.e., 1.2 log contrast) in 32 mild and 35 moderate ARMD eyes respectively [20]. Similarly, our present study reports a mean contrast sensitivity of 1.3 log contrast that ranged from 1.8 to 0.9 log contrast in 26 eyes with early and intermediate ARMD. Furthermore, our present study showed a median reading speed of 141 ± 45 words per minute with a range of 75 to 206 words per minute in eyes with early to intermediate ARMD. Likewise, Patel et al. reported an average maximum reading speed of 157 words per minute in eyes with dry and stable wet ARMD [25]. However, there was no previous research that reported the reading speed of early or intermediate ARMD separately.

Furthermore, in the present study, there was no noticeable difference found in the thickness and volume of total retina, outer retinal layer between early and intermediate ARMD. However, thickness and volume of retinal pigment epithelium-Bruch membrane complex and outer nuclear layer between early and intermediate ARMD were significantly different. Nevertheless, no previous study compared the OCT parameters between early and intermediate ARMD eyes. Thus, the present study results cannot be compared with previous researches. Karampelas et al. have reported average thickness of retinal pigment epithelium-Bruch membrane complex to be 22 micron in normal disease free eyes [5]. The present study showed average thickness of retinal pigment epithelium-Bruch membrane complex as 22.00 ± 3.61 and 26.57 ± 4.66 micron in early and intermediate ARMD eyes respectively. On the other hand, Matsumoto et al. (2009) reported average outer nuclear layer thickness at central fovea as 124.9 micron in normal eyes [26]. Menghini et al. reported average outer nuclear layer thickness as 82 ± 10 micron in normal eyes at foveal 0.5 degree whereas in the present study the average thickness of outer nuclear layer was recorded as 88.85 ± 3.80 and 70.89 ± 20.28 micron in early and intermediate ARMD eyes, respectively [27].

The major aim of this present study was to draw the correlation between visual functions and morphological parameters in eyes with early and intermediate ARMD. While assessing the relationship between visual functions and morphological parameters used, all the visual functions including distance visual acuity, near visual acuity, contrast sensitivity, and reading speed showed statistically significant correlation with average retinal pigment epithelium-Bruch membrane thickness, average outer nuclear layer thickness and average outer nuclear layer volume. However, reading speed showed highest correlation with average retinal pigment epithelium-Bruch membrane thickness, average outer nuclear layer thickness, average outer nuclear layer volume (*r* = 0.72, 0.60 and 0.69 respectively), followed by contrast sensitivity (*r* = 0.64) with average retinal pigment epithelium-Bruch membrane thickness. Moreover, contrast sensitivity showed significant association (*p* ≤ 0.05) with maximum numbers of OCT parameters used in the study including average retinal pigment epithelium-Bruch membrane thickness and volume, average outer nuclear layer thickness and volume. This indicates that reading speed and contrast sensitivity play an important role in representing the visual damage caused by early and intermediate ARMD. Thus, measuring these visual parameters along with high contrast distance visual acuity should be included in monitoring early and intermediate ARMD which otherwise is lacking with particular treatment strategy.

Furthermore, among the different quantitative retinal parameters used in the present study, both the average thickness and volume of outer nuclear layer were correlated significantly (*p* ≤ 0.05) with all of the visual functions. Outer nuclear layer is composed of cell bodies of rods and cones [28]. Thereby, outer nuclear layer being the component of photoreceptor, plays an important role in visual function and is considered to represent photoreceptor’s integrity [29,30]. Histopathological studies have confirmed loss of photoreceptors in ARMD eyes [9,31,32]. Sadigh et al. reported that outer nuclear layer is significantly thinner over drusen and thicker in para drusen areas [7]. Rogala et al. reported thinning of outer nuclear layer over drusen and paradrusen 150 um away from drusen [6]. Likewise, Schuman et al. reported thinning of photoreceptor layer over drusen in non-neovascular ARMD eyes [33]. Thus, previous researches have confirmed outer nuclear layer as one of the parameters affected by the disease process in early and intermediate ARMD. However, literature reporting the effect of outer nuclear layer loss on visual functions of ARMD eyes is significantly lacking. In our present study, visual functions including distance visual acuity and contrast sensitivity showed a good association with average outer nuclear layer thickness and volume (*r* ≥ 0.5) whereas reading speed showed a maximum correlation with average outer nuclear layer thickness and volume (*r* = 0.60, 0.69, respectively). Thus, the present study findings further support the fact that outer nuclear layer thickness and volume can be considered as an important biomarker in early and intermediate ARMD.

Among other OCT parameters used in the study, retinal pigment epithelium-Bruch membrane complex thickness is considered as one of the important site of alteration in early stages of ARMD. Similarly, Karampelas et al. have found a significant correlation between distance visual acuity and retinal pigment epithelium thickness (*r* = −0.363, *p* ≤ 0.05) in a retrospective study that included early to intermediate ARMD, but excluded geographic atrophy [5]. Likewise, Acton et al. reported significant correlation between retinal pigment epithelium thickening and worsening mean sensitivity (*r* = −0.448, *p* = 0.047) and mean deviation (*r* = −0.454, *p* = 0.045) of visual field using microperimeter [34]. They further stated that a 20% of the variance in retinal pigment epithelium thickness is attributed to the mean sensitivity and the mean deviation. Similarly, in our present study, retinal pigment epithelium-Bruch membrane thickness showed good correlation with distance visual acuity, near visual acuity, contrast sensitivity and reading speed (*r* = 0.55, 0.53, −0.64 and −0.72, respectively).

Previous researches have suggested that the primary changes in ARMD occur in Bruch membrane and retinal pigment epithelium, which eventually leads to damage to the adjacent photoreceptor components [9,35,36]. Aging changes that occur in Bruch membrane include accumulation of basal lamellar deposits and membranous debris, decreased elasticity, calcification and increase lipid concentration [37,38]. These changes all together thickens the Bruch membrane that acts as an extracellular matrix providing structural and biochemical support to the surrounding retinal pigment epithelium and choriocapillaris. Thus, dysfunction of Bruch membrane leads to weaken cell adhesion and anoikis-apoptosis [36]. Anoikis, the term used to describe apoptosis in response to inappropriate cell/or extra cellular matrix interactions, occurs when cells lose attachment to extra cellular matrix or adhere to an inappropriate type of extra cellular matrix [39]. Thus, in ARMD, anoikis-apoptosis occurs in retinal pigment epithelium and photoreceptor cells that use Bruch membrane as extra cellular matrix after Bruch membrane undergoes morphological alteration [36]. Furthermore, extracellular deposits of Bruch membrane trigger long-standing inflammation that might be a contributory factor in ARMD [36]. Thereby, retinal pigment epithelium and Bruch membrane being the primary site of ARMD development is justified to exhibit good correlation with visual functions in early to intermediate ARMD. Similarly, in the present study, a significant and negative correlation was found between average retinal pigment epithelium-Bruch membrane thickness and average outer nuclear layer thickness. This finding indicates that increase in retinal pigment epithelium-Bruch membrane thickness is in correspondence to the decrease in outer nuclear layer thickness.

However, outer retinal layer thickness was not significantly correlated with any of the visual functions. This can be because of the fact that outer retinal layer is summed of retinal pigment epithelium-Bruch membrane and photoreceptor’s outer and inner segment. While, retinal pigment epithelium- Bruch membrane is thickened [5,11], photoreceptors thickness decreases [7] in early to intermediate ARMD. Thus, these changes compensate each other to some extend when retinal pigment epithelium-Bruch membrane and photoreceptor’s outer and inner segment are measured together. Perhaps a separate measurement of photoreceptor inner and outer segment might be a more valid parameter. However, Spectralis auto segmentation software does not measure inner and outer segment of photoreceptors separately. This can be considered as one of the limitation of the study.

In addition, the present study has used the semi-automatic segmentation method while segregating the retinal layers of interest. The software incorporated in Spectralis OCT was employed first, followed by a manual correction of layer’s segmentation whenever required. Although past researches have used semi-automatic segmentation method for segregation of the retinal layers of interest in dry ARMD eyes [8,40], only a single study showed an inter observer agreement while assessing retinal nerve fiber layer, ganglion cell layer, and inner plexiform layer thickness (intra class correlation ≥ 0.85) [8]. The present study showed good inter-observer agreement of mean retinal pigment epithelium and Bruch membrane thickness, outer retinal thickness and outer nuclear layer thickness (intra class correlation ≥ 0.75) employing semi-automatic method of segmentation. Besides, a good association between the visual functions and retinal layer’s thickness was achieved with this segmentation method. Thus, the present study further strengthens the fact that semi-automatic methods of segmentation provides reliable measurement of retina layers in early to intermediate ARMD.

The present study has few limitations, the first being the inability of the automatic segmentation method to segregate the inner and outer segment of photoreceptor, which has been reported as one of the major site of changes to occur in the pathogenesis of ARMD [9]. Another constraint of the present study is the limited number of early ARMD eyes. As the study site was a referral center of ARMD, most of the eyes were intermediate ARMD. Furthermore, although the hospital is a tertiary eye center, it started its operation only in 2016. Thus, the numbers of patients seen at the ophthalmology department are comparatively low. These limitations could be addressed in future researches to further enhance our understanding on this aspect.

## 5. Conclusions

The present study suggests that reading speed, contrast sensitivity and near visual acuity along with distance visual acuity represent the morphological alteration in early and intermediate stages of ARMD and should be tested in clinical settings. Furthermore, the present study results strengthen the fact that rather than using total retinal thickness, average outer nuclear layer thickness and volume, and average retinal pigment epithelium-Bruch membrane thickness can be employed as important biomarkers while diagnosing early to intermediate ARMD.

## Figures and Tables

**Table 1:** Demographic profile of early and intermediate ARMD subjects.

Parameters	Early ARMD (*n* = 7)	Intermediate ARMD (*n* = 18)	Total (*n* = 25)
Age (mean ± S.D.)	61.00 ± 4.21 years	67.78 ± 5.62 years	65.96 ± 5.22 years
Gender	Number (percentage)	Number (percentage)	Number (percentage)
Male	2 (28.57%)	12 (66.66%)	14 (56%)
Female	5 (71.42%)	6 (33.33%)	11 (44%)
Ethnicity			
Malay	3 (42.85%)	5 (27.77%)	8 (32%)
Chinese	3 (42.85%)	11 (61.11%)	14 (56%)
Indian	1 (14.28%)	2 (11.11%)	3 (12%)
Distance visual acuity (mean ± S.D.)	0.05 ± 0.04 logMAR	0.36 ± 0.11 logMAR	0.28 ± 0.17 logMAR

S.D.: standard deviation.

**Table 2:** Descriptive statistics of visual functions in eyes with early and intermediate ARMD.

Visual Parameters	Early ARMD Mean/Median ± S.D.	Intermediate ARMD Mean/Median ± S.D.	Combined Early and Intermediate ARMD Mean/Median ± S.D.	Range
Mean distance visual acuity (logMAR)	0.05 ± 0.04	0.36 ± 0.11	0.28 ± 0.17	0 to 0.56
Mean near visual acuity (logMAR)	0.03 ± 0.05	0.32 ± 0.16	0.25 ± 0.20	0 to 0.6
Mean contrast sensitivity (log contrast unit)	1.59 ± 0.12	1.20 ± 0.24	1.30 ± 0.28	0.9 to 1.8
Mean reading speed * (wpm)	178	117	141 ± 45	75 to 206

S.D.: Standard deviation. *: Median. wpm: word per minute.

**Table 3:** Descriptive statistics of optical coherence tomography (OCT) parameters in eyes with early and intermediate ARMD.

OCT Parameters Mean ± S.D. (*n* = 7)	Early ARMD	Intermediate ARMD	Combined Early and Intermediate ARMD	Range
Average total retinal thickness (micron)	262.57 ± 17.33	257.36 ± 32.29	258.77 ± 28.78	205 to 297
Average total retinal volume (mm^3^)	0.21 ± 0.01	0.20 ± 0.25	0.202 ± 0.25	0.14 to 0.24
Average outer retinal layers thickness (micron)	89.28 ± 11.27	91.68 ± 7.60	91.04 ± 8.56	76 to 112
Average outer retinal layers volume (mm^3^)	0.06 ± 0.03	0.07 ± 0.06	0.068 ± 0.005	0.06 to 0.08
Average retinal pigment epithelium–Bruch membrane thickness (micron)	22.00 ± 3.61	26.57 ± 4.66	25.35 ± 4.80	17 to 34
Average retinal pigment epithelium-Bruch membrane volume (mm^3^)	0.01 ± 0.001	0.02 ± 0.006	0.02 ± 0.006	0.01 to 0.03
Average outer nuclear layers thickness (micron)	88.85 ± 3.80	70.89 ± 20.28	75.73 ± 19.13	34 to 102
Average outer nuclear layers volume (mm^3^)	0.07 ± 0.00	0.06 ± 0.015	0.06 ± 0.014	0.03 to 0.08

S.D.: Standard deviation.

**Table 4:** Correlation between visual parameters and quantitative OCT parameters.

Parameters	Average Outer Nuclear Layers Thickness	Average Outer Nuclear Layers Volume	Average Retinal Pigment Epithelium-Bruch Membrane Thickness	Average Retinal Pigment Epithelium-Bruch Membrane Volume	Average Total Retinal Thickness	Average Total Retinal Volume	Average Outer Retinal Thickness	Average Outer Retinal Volume
Distance visual acuity	*r* = −0.57 *p* = 0.003	*r* = −0.58 *p* = 0.002	*r* = 0.55 *p* = 0.004	*r* = 0.31 *p* = 0.122	*r* = −0.19 *p* = 0.363	*r* = −0.27 *p* = 0.184	*r* = −0.21 *p* = 0.297	*r* = −0.05 *p* = 0.792
Near visual acuity	*r* = −0.45 *p* = 0.020	*r* = −0.51 *p* = 0.008	*r* = 0.53 *p* = 0.006	*r* = 0.13 *p* = 0.010	*r* = −0.02 *p* = 0.939	*r* = −0.10 *p* = 0.618	*r* = −0.19 *p* = 0.355	*r* = −0.12 *p* = 0.565
Contrast sensitivity	*r* = 0.52 *p* = 0.006	*r* = 0.53 *p* = 0.005	*r* = −0.61 *p* = 0.000	*r* = −0.43 *p* = 0.027	*r* = 0.25 *p* = 0.223	*r* = 0.31 *p* = 0.129	*r* = −0.30 *p* = 0.137	*r* = −0.01 *p* = 0.964
Reading speed *	*r* = 0.60 *p* = 0.006	*r* = 0.69 *p* = 0.001	*r* = −0.72 *p* = 0.000	*r* = −0.14 *p* = 0.571	*r* = 0.03 *p* = 0.900	*r* = 0.24 *p* = 0.347	*r* = −0.18 *p* = 0.476	*r* = 0.24 *p* = 0.344

*r*: Correlation coefficient. *p*-values are statistically significant (*p* < 0.05). *: Spearman’s correlation.
